# Supplementary material for: Effects of process parameters on capsule size and shape in the centrifugal encapsulation technology: Parametric study dataset
Source: Data Brief. 2022 Jan 20;41:107851. doi: 10.1016/j.dib.2022.107851 (PMC8804192; doi:10.1016/j.dib.2022.107851)
Supplement: Supplementary file 1 [file mmc1.zip › Word/article_dib_doc.docx]

**Article information**

**Article title**

Effects of Process Parameters on Capsule Size and Shape in the Centrifugal Encapsulation Technology: Parametric Study Dataset

**Authors**

Matei Badalan^1^, Frederic Bottausci^1^, Giovanni Ghigliotti^2,*^, Jean-Luc Achard^2^, Guillaume Balarac^2^

**Affiliations**

^1^ Univ. Grenoble Alpes, CEA, LETI, Technologies for Healthcare and biology division, Microfluidic Systems and Bioengineering Lab, 38000 Grenoble, France

^2^ Univ. Grenoble Alpes, CNRS, Grenoble INP, LEGI, 38000 Grenoble, France

**Corresponding author’s email address and Twitter handle**

Tel.: +33 (0)4 76 82 51 70

Email: giovanni.ghigliotti@univ-grenoble-alpes.fr

**Keywords**

Microencapsulation; centrifugal microfluidics; alginate hydrogels; cell therapy; cell encapsulation

**Abstract**

Microencapsulation technologies have experienced much growth over the past decades and are commonly used for food, cosmetic, pharmaceutical and biomedical applications. Certain application fields impose stricter requirements on the polymer capsules. In many biomedical applications including bioencapsulation, cell therapy and drug delivery applications, capsules are required to have a controlled shape and size, as well as a defined mechanical stability and porosity.

This data article reports the alginate capsule production using common centrifugal technology, which enables the production of microcapsules with highly viscous biopolymers. We describe the experimental data generated in a parametric study, where the main control parameters of the centrifugal encapsulation system (alginate viscosity, rotating speed, nozzle diameter, collecting distance) were varied. The geometric properties of the produced hydrogel capsules were analysed by microscope photography and image processing. The dataset presented here contains the experimental data, the raw capsule images, the analysis scripts, the analysed images, and tables with extracted geometric information. All extracted data was compiled into a table containing geometric properties of more than 50000 analysed capsules.

These data allow (i) to reproduce quickly the encapsulation experiments and be able to choose in a straight-forward manner the combination of parameters needed in order to generate capsules with desired properties; (ii) to create more general phase diagrams of the centrifugal encapsulation technology which can be widely used for prediction and/or parameter selection; (iii) to analyse more thoroughly the sensitivity of capsule properties to given stages of the encapsulation process.

The research article on these data [1] was published in the journal Colloids and Surfaces A: Physicochemical and Engineering Aspect, with the title: Three-dimensional phase diagram for the centrifugal Calcium-alginate microcapsules production technology.

**Specifications table**

| **Subject** | Chemical Engineering: Colloid and Surface Chemistry |
| --- | --- |
| **Specific subject area** | Centrifugal (micro-)encapsulation technology |
| **Type of data** | Table  Image  Figure |
| **How the data were acquired** | Instruments: IX50 inverted microscope, BX60 microscope (Olympus, Japan)  Software: Cellprofiler 4 image analysis software (Broad Institute, USA) |
| **Data format** | Raw  Analyzed |
| **Description of data collection** | Centrifugal capsule production experiments were carried out using different combinations of input parameters. After hardening in the gelation solution, the generated microcapsules were collected and placed in Petri dishes for microscope observation. A digital camera connected to an inverted microscope was used to collect photos of the capsules. The collected images were analysed using CellProfiler 4 image processing software, in order to detect the objects (capsules) and measure their geometric properties. |
| **Data source location** | Institution: CEA LETI  City/Town/Region: Grenoble  Country: France  Latitude and longitude for collected samples/data: 45.201106, 5.705566 |
| **Data accessibility** | Repository name: Effect of process parameters on the shape and size of Calcium-alginate microcapsules generated with the centrifugal encapsulation technique – Main  Data identification number: 10.17632/8spjcc6r69.1  Direct URL to data: https://data.mendeley.com/datasets/8spjcc6r69/draft?a=988ef051-8a58-4336-b6bc-54eb01bd455c  Instructions for accessing these data: The data is accessible using the provided link. The raw data has been split in several Mendeley Data repositories due to the limit of 10GB per repository. The links to all the related raw data repositories are listen in the main repository (see link above). |
| **Related research article** | M. Badalan, F. Bottausci, G. Ghigliotti, J.L. Achard, G. Balarac, Three-dimensional phase diagram for the centrifugal Calcium-alginate microcapsules production technology, Colloids and Surfaces A: Physicochemical and Engineering Aspects. In Press. |

**Value of the data**

• The data described here represents a novel and extensive exploration of the effects of the four most relevant control parameters of the centrifugal encapsulation technology (alginate viscosity, needle diameter, collecting distance, rotating speed) on the properties (shape, size) of the produced capsules, over a wide range of values. This parametric study allows building prediction models, which render all users of this technology able to precisely control the output of the process by choosing the appropriate input parameters.

• This data can be used by anyone who wishes to implement a centrifugal encapsulation device, in order to be able to predict the properties of the capsules based on the control parameters or to select the control parameters needed for a desired output.

• This dataset and the method described here give all the information needed to quickly implement a centrifugal encapsulation technology, adapt it to a given equipment and be able immediately to choose the optimal operating points, without the need of a long trial-and-error process. Given the fact that 4 main control parameters were varied over large ranges of values, this dataset can also be used in order to understand more thoroughly the physics of the centrifugal encapsulation process and the effects of each phenomenon involved in the process on the properties of the produced capsules.

• The dataset contains additional information regarding the 3D shape of the produced capsules, which is often an important issue when analysing items based on 2D images.

• The dataset contains a vast amount of capsule images in various shapes and features. Additionally, it contains labels which were set by visual observation in order do describe the shape family of the capsules produced in each experimental point. These images can be used as a training dataset to develop more efficient methods of capsules characterisation by image analysis.

**Data description**

This data represents raw and analysed results of the parametric study on centrifugal capsule production. The results of 201 different capsule production experiments are presented. The results represent the properties (size, shape) of the produced capsules.

In the main dataset, the file ***Experimental_design.csv*** contains a table with the values of the 4 control parameters in each of the experiments. The experiments are numbered according to their order in the experimental campaign (which contained 254 experiments). From the original experimental campaign, 53 of the experiments had an auxiliary purpose and were carried out with alginates that were not characterised. These experiments are not presented here. Only the remaining 201 experiments are described here, but the experiment numbering was kept as in the original campaign (from 1 to 254). Two additional columns, *3D_analysis* and *fibre*, indicate whether a separate analysis of the 3D capsule shape was performed in that experimental point, or whether separate photos of a produced fibre are available for that experimental point.

Table ***Microscope_pixel_mm_conversion.xlsx*** gives the conversion factors from pixels to millimetres on the microscopic images taken with the microscopes used for capsule characterisation. These conversion factors were obtained while calibration the microscopes using standard microscope calibration slides.

The folder ***Material properties*** contains a table with the measured properties of the alginate solutions (zero-shear viscosity, density, surface tension) and a subfolder where the viscosity measurement curves (shear viscosity versus shear rate) of the different alginate solutions are given in separate tables. The figure ***Viscosity alginates.jpg*** is a plot of these curves in logarithmic scale.

The folder ***samples*** contains two microscope images of capsules produced in each of the 201 experimental points. These can be used to give a quick qualitative insight of the properties of capsules generated in each operating point.

In the datasets linked to the main dataset, each folder ***Exp_i*** contains the raw microscope images (***.tif*** files) of capsules produced in the experiment number ***i***. Each image name contains the tube position (for example *t1*), the microscope magnification used when taking that image (for example *2.5X*) and the date and time when the photo was taken. In addition, each folder contains the image analysis (CellProfiler 4.0) script which was used to analyse the images, under the name ***pipelineMX.cppipe***, where *M* is the microscope magnification of the image series. A different image analysis script was used per experiment and per magnification. The image files with the suffix *_Objects.tiff* represent the analysed images, where the capsules detected by the image analysis software are marked with red contours. Each raw image has a corresponding analysed image in the same folder. Finally, each image analysis script generates 4 ***.csv*** files with the prefix *Properties* and the magnification included in the file name. The file ***Properties_MX_Experiment.csv*** gives a short summary of the analysis run by the image processing software, including the pipeline used. File ***Properties_MX_Image.csv*** contains a summary of the images that were analysed by the software and the average of the properties of the capsules measured per image. File ***Properties_MX_RawCapsules.csv*** contains all the raw capsules detected by the corresponding script, including the metadata extracted from the image file name and the values of measured geometrical properties of each capsule. File ***Properties_MX_FilteredCapsules.csv*** contains the same information as the previous one, but only the capsules that passed the filter, based on the value of the solidity, are preserved.

If in a certain experiment number *i* a fibre was observed in the gelation bath, a few photos of this fibre were taken and placed inside the subfolder named ***fibre*** of the folder ***Exp_i***.

If in a certain experiment *i* an additional 3D analysis of the capsule shapes was performed, a subfolder called ***3D_analysis*** was created inside folder ***Exp_i***, where the raw images, the *.cppipe* script, the analysed images and the image analysis tables are placed.

In the main dataset, the table ***Qualitative_properties.csv*** contains supplementary properties of the capsules which were collected by visualisation of a few images per each experiment and labelled manually according to a certain code. The labels do not describe individual capsules, but rather average qualitative properties of the capsules formed in a given operating point.

- Column *Quality* describes the quality (integrity) of the capsule surface, which was coded with the following 4 levels, 0: smooth capsule surface without visible damage; 1: surface with little damage or wrinkles; 2: damaged surface with the formation of a "skirt" or high surface irregularities; 3: totally broken or burst capsules.
- Column *Filament* indicates the presence (value 1) or absence (value 0) of thin gelled filaments or cordlets attached to the capsule.
- Column *Fibre* indicates the presence or not of a long fibre in the gelation bath, with the following classes, 0: no fibre observed; 1: fibre fragments observed; 2: a long (often entangled) fibre observed.
- Column *Satellites* describes approximately the ratio between the number of satellite capsules and the number of main capsules observed, 0: ratio less than 1/3, 1: ratio between 1/3 and 3, 2: ratio greater than 3.
- Column *Shape Family* represents a manual qualitative classification of the capsule shapes in one of the three families: ellipsoidal, tear-shaped (*tear*) or deformed. This manual, qualitative labelling is not to be mistaken with the automated quantitative capsule shape classification method presented in [1].

The folder ***Examples 3D analysis*** gives a few examples of images taken in the 3D capsule shape analysis, for each type of capsule shape. The image analysis files are not given, as these should only serve as examples of the 3D shape analysis method.

The Python script ***gather_all_filtered_capsules.py*** (uses Python 3 and requires the packages Pandas and Numpy) was used to concatenate all the tables with properties of the filtered capsules from the subfolders (excluding the 3D image analysis tables) into an unique table ***Filtered_capsules_dataset.csv***. Additionally, the units of all the geometric measurements having the size of a length, area or volume were converted from pixels (as exported by the image analysis software) into mm, using the conversion factors stored in table ***Microscope_pixel_mm_conversion.xlsx***. The script then joins the tables of the experimental design and of qualitative properties to this table in order to create one dataset containing at the same time all the experimental data, the qualitative properties of the capsules and the extracted geometric measurements. **This table, named *Complete_filtered_capsules_dataset.csv* is the core result and comprises the most important data of this experimental campaign. The table contains one entry per each capsule detected, and provides at the same time all the information on how the capsule was produced and the characterisation (both quantitative and qualitative) of the capsule size and shape.** The table contains information on more than 50 000 capsules, that were produced in our experiments.

The Python script ***mean_properties.py*** (uses Python 3 and requires the packages Pandas and Numpy) reads the previously created tables and creates a table with the summary of the obtained results, ***Capsule_properties_mean_and_std.csv***. This table contains one entry per each experiment. Each entry contains the parameters characterising that operating point, the qualitative properties of the capsules produced in that operating point, and the mean and standard deviation of the geometric measurements of the capsules analysed in that operating point.

Finally, an example of data plotting script (under the form of a Jupyter Notebook, ***Data_plotting.ipynb***) is given in the main dataset. It extracts the data from the previously created tables and draws plots of capsule properties based on the input parameters. The following figures, representing the produced data, were plotted using this script.

Figure 1 represents a colour plots of capsule diameter^[[1]](#footnote-2)^ depending on two input parameters, the nozzle diameter and the rotation speed.

Figure 2 represents colour plots of 4 different capsule shape properties^[[2]](#footnote-3)^ depending on alginate concentration and collecting distance.

The folder ***Data plots*** in the main dataset contains the figures created by this script.

*
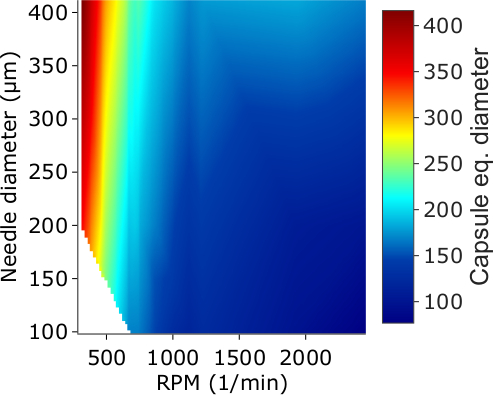
*

Figure 1. Representation of the data: capsule diameter as a function of nozzle diameter and rotation speed.

| 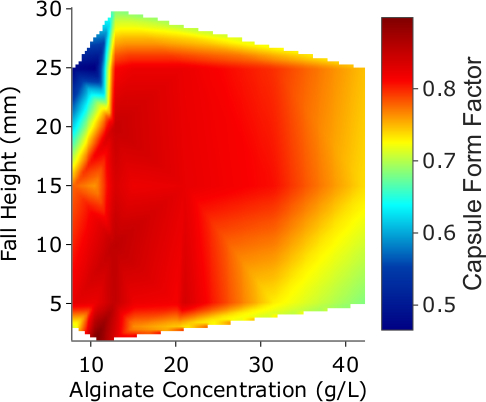  a) Form factor | 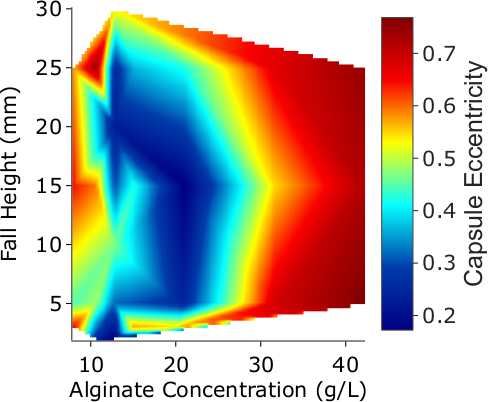  b) Eccentricity |
| --- | --- |
| 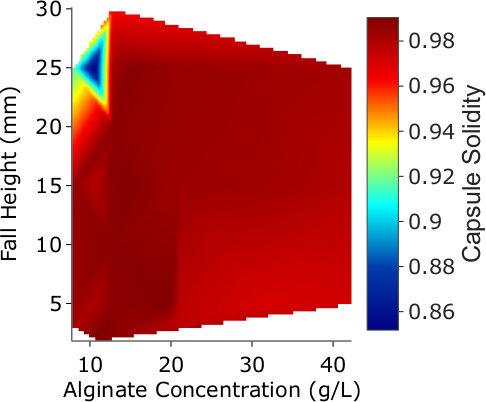  c) Solidity | 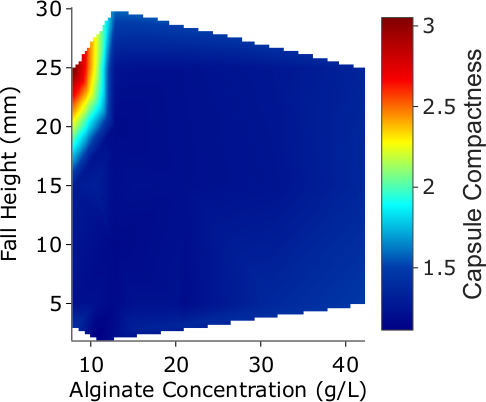  d) Compactness |

Figure 2. Representation of the data: capsule shape properties as functions of alginate concentration and fall height.

**Experimental design, materials and methods**

*Materials*

The alginate solution used in the experiments is prepared from a highly purified sodium alginate basis, PRONOVA SLG100 (Novamatrix, Norway), commonly used in bio-encapsulation applications. All alginate solutions used in this study were prepared from alginate originated from the same batch. The lyophilised alginate powder is diluted with an alginate buffer solution, containing microfiltered water, 150mM NaCl and 25mM HEPES^[[3]](#footnote-4)^, to produce sodium alginate solutions of different concentrations. In practice, usually first a solution of 40 g/L is prepared and stirred over 24h. After this, solutions of different concentrations are obtained by combining the 40 g/L solution with alginate buffer in different proportions.

The gelation bath is prepared using microfiltered water, 80mM NaCl, 100mM CaCl_2_ and 25mM HEPES buffer^[[4]](#footnote-5)^. The The NaCl and CaCl_2_ powder were purchased from Sigma-Aldrich (USA) and the HEPES was purchased from Fisher scientific (France). The CaCl_2_ concentration in the gelation solution is 100 mM, as it is typically used for Ca-alginate bead formation [4]. The Ca concentration in the gelation bath was kept constant in all our experiments. As opposed to common practice in general encapsulation procedures [4], no surfactant was added to the gelation solution in our experiments. This choice was motivated by the fact that surfactants may have an inhibitory effect on the encapsulated cells or biomaterials in biomedical encapsulation applications.

*Physical properties of solutions*

The shear viscosities of the solutions are measured using a KINEXUS pro+ cone and plate rheometer from Malvern Instruments Limited (UK). The viscosities are measured over a range of shear rates from 10^-1^ s^-1^ to 10^3^ s^-1^. The value of viscosity that we give in the experimental design tables as a control parameter of the process is the zero-shear viscosity of the alginate solutions.

The surface tension of the gelation bath was measured using the pendant drop method and found to be 71.91 mN/m. The surface tensions of four of the alginate solutions were measured with the same method.

The density of the alginate buffer was measured using a pipette and a precision balance. Only for one concentration (21 g/L), the density of the alginate solution was measured, with 5 replicates. The density values of the other alginate solutions were calculated based on the mixture law, which predicts the density of a diluted solution $\rho_{alg,dil}$ based on the density of the alginate buffer $\rho_{buff}$, the density of the precursor concentrated alginate solution $\rho_{alg,conc}$ and the volumes of each of them that were used:

$$\rho_{alg, dil}=\frac{\rho_{alg,conc}V_{alg,conc}+\rho_{buff}V_{buff}}{V_{alg,conc}+V_{buff}}$$

*Device description*

The experimental device used in to generate the data is an adaptation of the device presented in [5]. A schematic representation of the device is given in figure 4. It is composed of a 50ml FALCON centrifuge tube, a needle holder (own design and fabrication, drawings given in figure 5) and a dispensing needle (Nordson EFD Optimum general purpose dispensing tips), which consists of a small reservoir and a stainless steel capillary. Needles with inner diameters of 100, 150, 200, 330 and 410 $\mu m$ were used. The length of the needle was 12.7 mm for all needle sizes, except for the 100 $\mu m$ needle, which has a length of 6.35 mm. Figure 5 shows drawings of the needle holder used in our experiments, for both needle lengths 12.7mm (a) and 6.35mm (b). The hole in the holder for the 6.35 mm needles was chamfered at the bottom. The needle holders were manufactured using transparent polycarbonate.

The centrifuge tube is placed in a conventional laboratory centrifuge of type Eppendorf 5810 R (Germany) (figure 3) equipped with a swing bucket rotor of type A-4-62. Figure 4 also comprises some of the most important geometrical parameters of the device: *y_tip_*, *h*, *D_tube_* and the centrifugal force. The distance from the centrifuge rotation axis to the needle tip, denoted *y_tip_,*  and the FALCON tube diameter, *D_tube_* are kept constant all along our experiments. The height of fall of the droplets *h* is therefore controlled solely by the quantity of gelation solution filling the centrifuge tube. In our experiments, *y_tip_* was measured to approximately 81 mm and the inner tube diameter of the FALCON centrifuge tube is 27.8 mm.


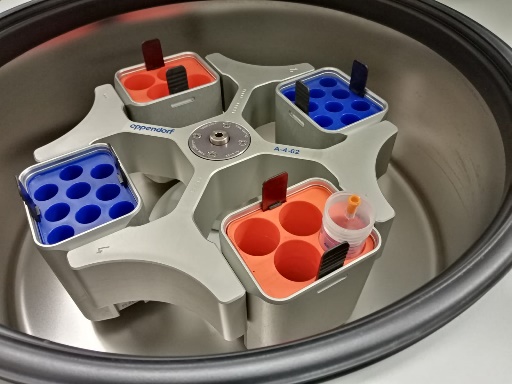


Figure 3. Image of the centrifuge used in the experimental campaign. Only the 2 orange swing buckets were used, in order

**
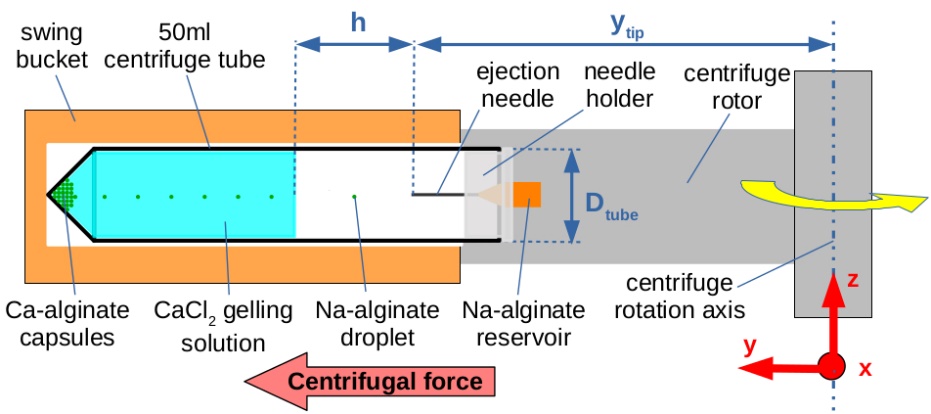
**

Figure 4. Description of the centrifugal encapsulation (CENTRICAPS) device. The 50 ml centrifuge tube, filled with gelation solution and equipped with a needle holder at the top, is placed in a swing bucket centrifuge. Under the effect of the centrifugal force, the Na-alginate solution flows from the reservoir, through the capillary and is ejected under the form of droplets from the needle tip. The droplets fly through the air and impact the gelation solution, where they start gelling and sediment on the bottom due to a slight density difference with the solution.

| **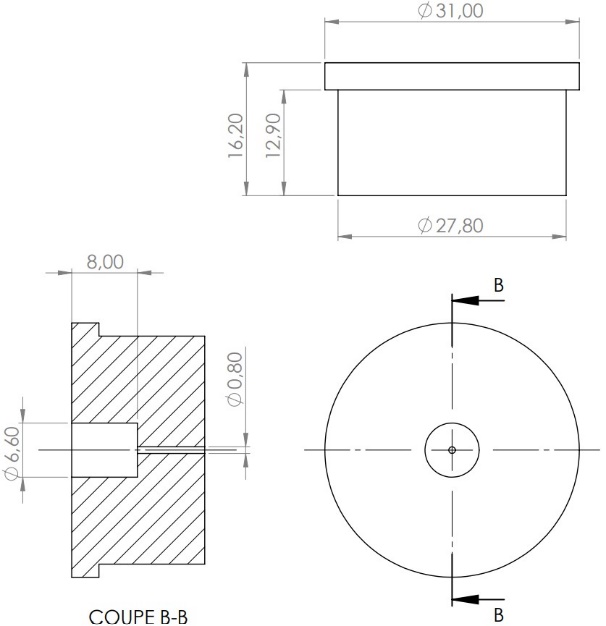**  **a**) | **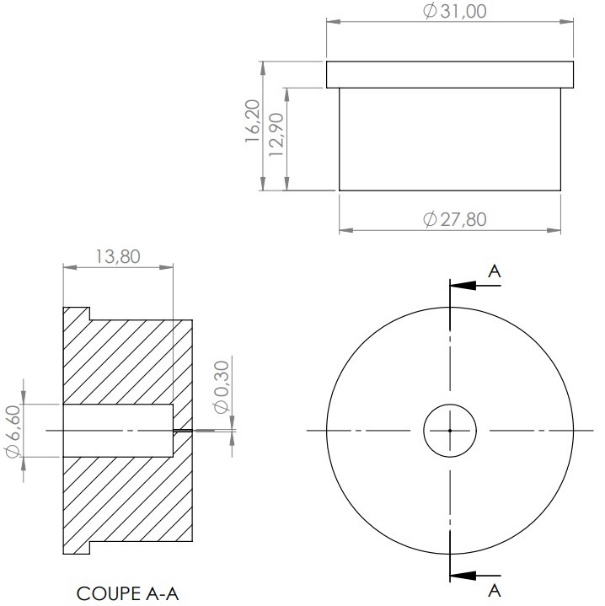**  **b**) |
| --- | --- |

Figure 5. Drawings of the needle holder for needle lengths of 12.7mm (left) and 6.35mm (right).

The working principle of this device is the following: under the effect of the centrifugal force, the Na-alginate solution flows from the reservoir into the capillary and is ejected from the capillary tip under the form of droplets. These droplets fly through air in the centrifugal field and enter into the gelation bath. Due to the presence of Ca ions in the gelation bath, the cross-linking reaction of the alginate chains with the Ca ions takes place. The droplet gelation takes place as it sinks in the solution (due to a slight density difference) and sediments on the bottom. The thus formed Ca-alginate capsules accumulate on the bottom of the centrifuge tube.

*Experimental procedure*

The centrifuge tube is filled with gelation solution up to a certain level, according to the desired fall height of the droplets *h* (collecting distance). The needle reservoir is filled with Na-alginate solution. Typically, between 100 $\mu l$ and 20 $\mu l$ were used for each needle in our experiments. Then, the needle is introduced in the needle holder. The needle holder is then fixed at the top of the FALCON centrifuge tube, and the tube is placed in the centrifuge. The centrifuge is balanced, the desired rotation speed and centrifugation time are selected and the centrifugation is started.

The centrifugation time should be chosen preferably large enough so that all the Na-alginate solution is evacuated from the reservoir and ejected from the needle. Otherwise, there is a danger of ejecting some unwanted droplets during the transient deceleration phase of the centrifuge, when the rotation speed (and thus the centrifugal force) is lower than the nominal one. These droplets will then have a size significantly higher than that of the majority of the droplets ejected during the centrifugation at nominal rotating velocity, thus affecting the size monodispersity of the formed capsules. The centrifugation time for 10-20 $\mu l$ of alginate solution, in order to obtain a sufficient amount of capsules for analysis, can range from a few minutes for high rotation velocities, high needle diameters and low alginate viscosities, to about 20-25 minutes for low rotation velocities, low needle diameters and high alginate viscosities.

Finally, the value of the temperature to be maintained constant inside the centrifuge during the centrifugation process was selected to be 20° C in all our experiments.

Once the centrifugation is over, the tube is collected and the needle holder is taken out. The tubes are left for at least 5 to 10 minutes in order to let all remaining capsules settle on the bottom of the tube and to give enough time for the hardening of the capsules [6]. It should be kept in mind that the typical capsule diameter produced in our experiments is of the order of a few hundreds of $\mu m$, compared to diameters of a few mm as in gravitational dripping. Because of the smaller size, the necessary time for the diffusion of Ca ions inside the alginate capsule and thus for the hardening of the gel is expected to be shorter.

After the centrifugation, the Ca-alginate capsules are located at the bottom of the tube, due to their density which is slightly higher than that of the aqueous gelation solution. The supernatant from the FALCON centrifuge tube is extracted with a pipette, leaving around 10ml of solution on the bottom of the tube. The remaining liquid is first stirred, either by repeated blowing with a pipette or by gently shaking or rotating the tube, in order to put the alginate capsules in suspension in the liquid. This solution is then poured into a Petri dish. Typically, Petri dishes with a diameter of 6 cm were used. The level of liquid in the Petri dish is of around 3 to 5 mm, which is around 10 times the typical size of the capsules. By waiting a few seconds, we ensure that the capsules are located on the bottom of the dish and covered by a sufficiently deep layer of liquid, avoiding any free surface effects. The Petri dish is then covered with its transparent lid and placed under microscope for observation.

The data for the analysis of capsule sizes and shapes is collected using a digital camera connected to an inverted microscope (Olympus IX50, Japan)^[[5]](#footnote-6)^, using lens with a magnification varying from 2.5x to 10x, chosen conveniently depending on the size of the beads. The focus of the lens is adjusted in order to capture clearly the contour of the capsules. Then, the image of the bottom plane of the Petri dish is swept manually using the microscope, in order to take photos of the capsules in the Petri dish, using the digital camera connected to the microscope. Around 15-20 photos are taken for each Petri dish with a low-zoom lens (typically 2.5x), and 5-10 complementary photos are taken with a higher zoom (typically 4x). The number of capsules captured in an image can vary from 5 to 50, depending on the size of the capsules and the lens chosen. **The 2D images taken represent the raw data of our experiments**.

A previous calibration of the digital camera and microscope has been performed, using standard microscope calibration slides. For each lens used, a conversion factor has been obtained, allowing to convert the length from pixels to mm.

In addition to the main experimental campaign, in order to investigate the three-dimensional shape of the capsules, we performed a complementary analysis, where single capsules, located inside the Petri dish with gelation solution, were turned manually using a needle and photos were taken from various sides. This analysis is important in order to avoid certain wrong hypothesis that can be made on the third dimension of objects when analysing them only from 2D images.

*Design of experiments*

In this study, four main operating parameters are varied (see table 1): the alginate viscosity $\eta_{alg}$, the inner diameter of the ejection needle $D_{needle}$, the centrifuge rotating speed $\omega$ and the quantity of gelation solution in the centrifuge tube, which controls the fall height of the droplets $h$. A summary of the experimental design is given in table ***Experimental_design.csv*** in the dataset related to this article. Around 110 experiments (distinct sets of the 4 parameters) were realised in triplicate. Other 90 supplementary operating points were realised in unicate.

Table 1. Summary of the main operating parameters varied in this study, with ranges of variation..

| Parameter | Minimum value | Maximum value |
| --- | --- | --- |
| $\eta_{alg} (Pa.s)$ | 0.078 | 18.556 |
| $D_{needle}(\mu m)$ | 100 | 410 |
| $\omega(RPM)$ | 315 | 2440 |
| $h (mm)$ | 2 | 25 |

For the 110 experiments performed in triplicate, the three samples were prepared using different tubes, supports and needles, and the solutions were handled using different pipettes, in order to avoid contamination. The 3 samples were centrifuged in the same run, by placing the three tubes in different positions in the centrifuge (see figure 6). The tubes were numbered (1,2 or 3) and the tube number was recorded along with the experimental data and the results. The capsule images are marked according to the tube number, so that exact information about the origin of each capsule can be found if needed. For the experiments performed in unicate, only position 1 was used. In the experiments performed in triplicate, tubes 1 and 3 were placed in positions P1 and P2 (figure 6) of a swing bucket, whereas tube 2 and the tare tube were placed in the positions P1 and P2 of the opposite swing bucket.


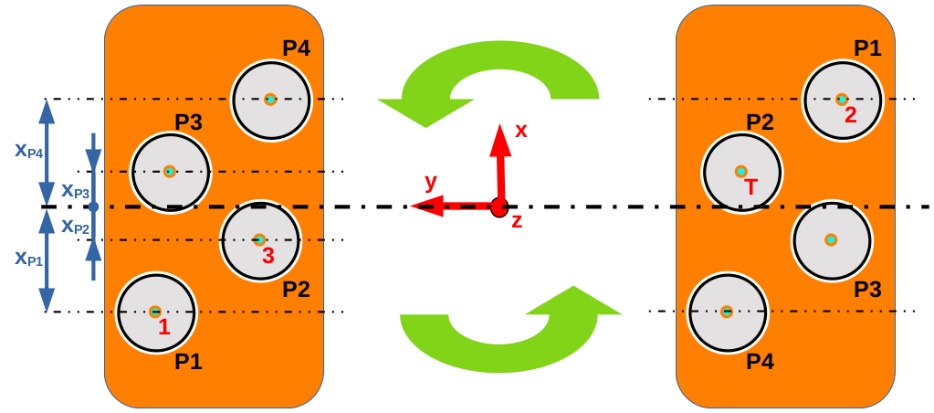


Figure 6. Schematic top view of the 2 swing buckets for 50mL centrifuge tubes, when the centrifuge is at rest. The green arrows indicate the rotation direction when the centrifugation is started. The 4 different tube positions in each swing bucket are numbered P1,P2,P3 and P4. Their respective distances to the y-z plane, which contains the rotation axis and passes through the middle planes of the two swing buckets, are indicated with arrows on the left side of the drawing. The red labels 1,2, 3 and T indicate the position of the three tubes and of the tare tube in the experiments which were realised in triplicate.

The distance from the axis of the centrifuge tube to the y-z plane is marked in figure 6 as xP1, xP2, xP3, xP4. The corresponding values are xP1=xP4 = 24 mm; xP2=xP3=11 mm. It is recommended to choose *h* smaller than 30 mm for tube positions P1 and P2 and choose *h* smaller than 15 mm for tube positions P3 and P4.

For series of 110 experiments realised in triplicate, an analysis has been carried out to identify the degree of agreement between the results obtained with the different tubes 1,2 and 3 (see figure 7). This was assessed by comparing the statistical distributions of a size indicator (the equivalent diameter) and of a shape indicator (the form factor) for each of the 3 tubes. This comparison was repeated for various operating points. The mean values of these indicators obtained with the 3 tubes were found to lie within less than 34% (standard deviation) distance from one another. Moreover, no systematic errors between the 3 tubes have been observed. This good agreement in the results justified the usage of unicate samples for further experimental points.

| **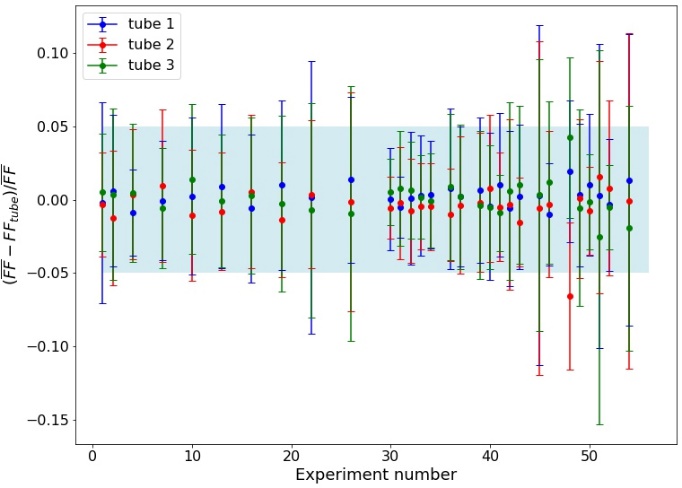**  **a**) | **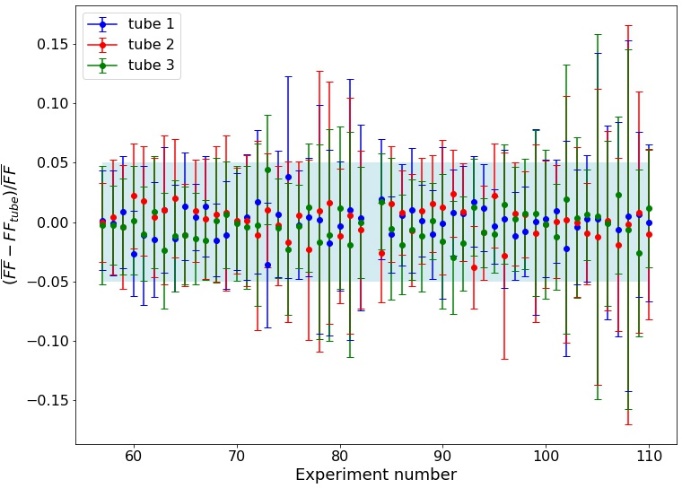**  **b**) |
| --- | --- |
| **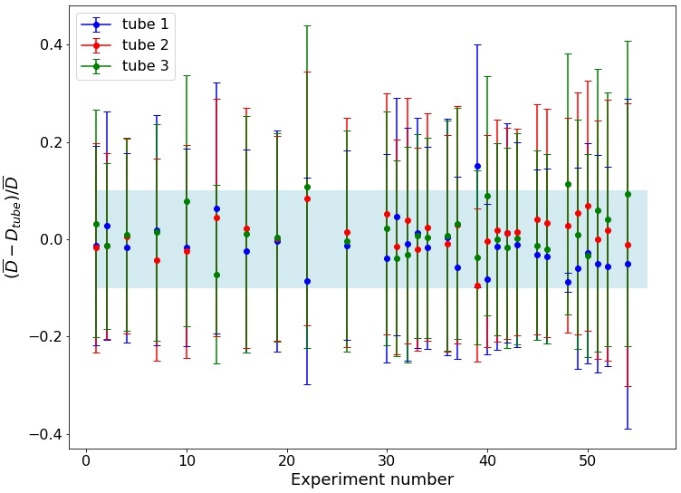**  **c**) | **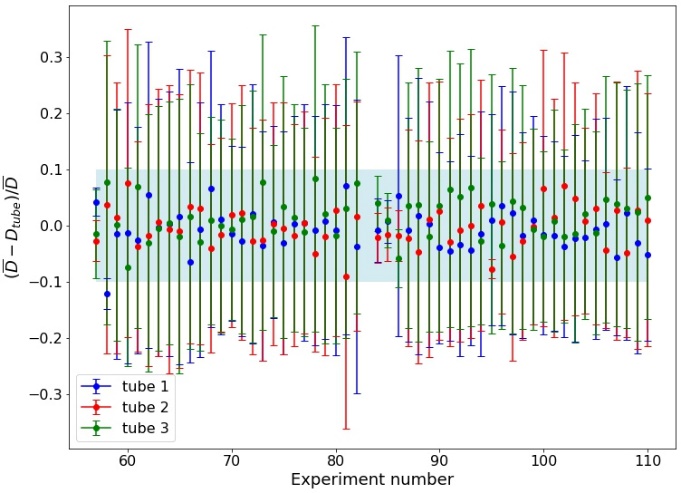**  **d**) |

Figure 7. (a) and (b): distributions of the capsule form factors $FF_{tube}$ obtained in each experiment using each of the three different tubes. The distributions are normalised with respect to the mean value of the form factor in that experiment, $\bar{FF}$. The blue region represents the $\pm5\%$ region. (c) and (d): distributions of the capsule equivalent diameters $D_{tube}$ obtained in each experiment using each of the three different tubes. The distributions are normalised with respect to the mean value of the equivalent diameter in that experiment, $\bar{D}$. The blue region represents the $\pm10\%$ region.

**CRediT author statement**

**Matei Badalan:** Investigation, Formal analysis, Visualization, Writing - Original Draft

**Frederic Bottausci:** Conceptualization, Methodology, Validation

**Giovanni Ghigliotti:** Conceptualization, Validation, Writing - Review & Editing

**Jean-Luc Achard:** Validation, Writing - Review & Editing, Supervision

**Guillaume Balarac:** Supervision

**Acknowledgments**

This research did not receive any specific grant from funding agencies in the public, commercial, or not-for-profit sectors.

**Declaration of interests**

The authors declare that they have no known competing financial interests or personal relationships that could have appeared to influence the work reported in this paper.

**References**

[1] M. Badalan, G. Ghigliotti, F. Bottausci, J.-L. Achard, G. Balarac, Phase Diagram for the Centrifugal Calcium Alginate Microcapsules Production Technology, Colloids and Surfaces A (2022).

[2] Measurement — CellProfiler 4.0.7 documentation, 2021. URL: https://cellprofiler-manual.s3.amazonaws.com/CellProfiler-4.0.7/modules/measurement.html, accessed: 2021-04-20.

[3] J. A. De Lora, J. L. Velasquez, N. J. Carroll, J. P. Freyer, A. P. Shreve, Centrifugal Generation of Droplet-Based 3D Cell Cultures, SLAS Technology 25 (2020) 436–445.

[4] B. B. Lee, P. Ravindra, E. S. Chan, Size and shape of calcium alginate beads produced by extrusion dripping, Chemical Engineering and Technology 36 (2013) 1627–1642.

[5] S. Haeberle, L. Naegele, R. Burger, F. V. Stetten, R. Zengerle, J. Ducree, Alginate bead fabrication and encapsulation of living cells under centrifugally induced artificial gravity conditions, Journal of Microencapsulation 25 (2008) 267–274.

[6] P. Smrdel, M. Bogataj, A. Mrhar, The influence of selected parameters on the size and shape of alginate beads prepared by ionotropic gelation, Scientia Pharmaceutica 76 (2008) 77–89.

1. Equivalent diameter extracted by the image processing software [2]. [↑](#footnote-ref-2)
2. Form factor, solidity, eccentricity and compactness, cf. [2] for definitions. [↑](#footnote-ref-3)
3. N-2-Hydroxyethylpiperazine-N-2-Ethane Sulfonic Acid. [↑](#footnote-ref-4)
4. The presence of NaCl and HEPES buffer is important when encapsulating cells, in order to ensure buffering capacity and osmotic balance, and thus biocompatibility of the solutions [3]. Since the work here try to reproduce the conditions of bioencapsulation experiments, the solutions were formulated keeping in mind the requirements for bioencapsulation applications. [↑](#footnote-ref-5)
5. In few of the experiments, a microscope Olympus BX60 was used for analysis. [↑](#footnote-ref-6)
