# Supplementary material for: Effects of process parameters on capsule size and shape in the centrifugal encapsulation technology: Parametric study dataset
Source: Data Brief. 2022 Jan 20;41:107851. doi: 10.1016/j.dib.2022.107851 (PMC8804192; doi:10.1016/j.dib.2022.107851)
Supplement: Supplementary file 1 [file mmc1.zip › Word/images.zip!/compactness.pdf]

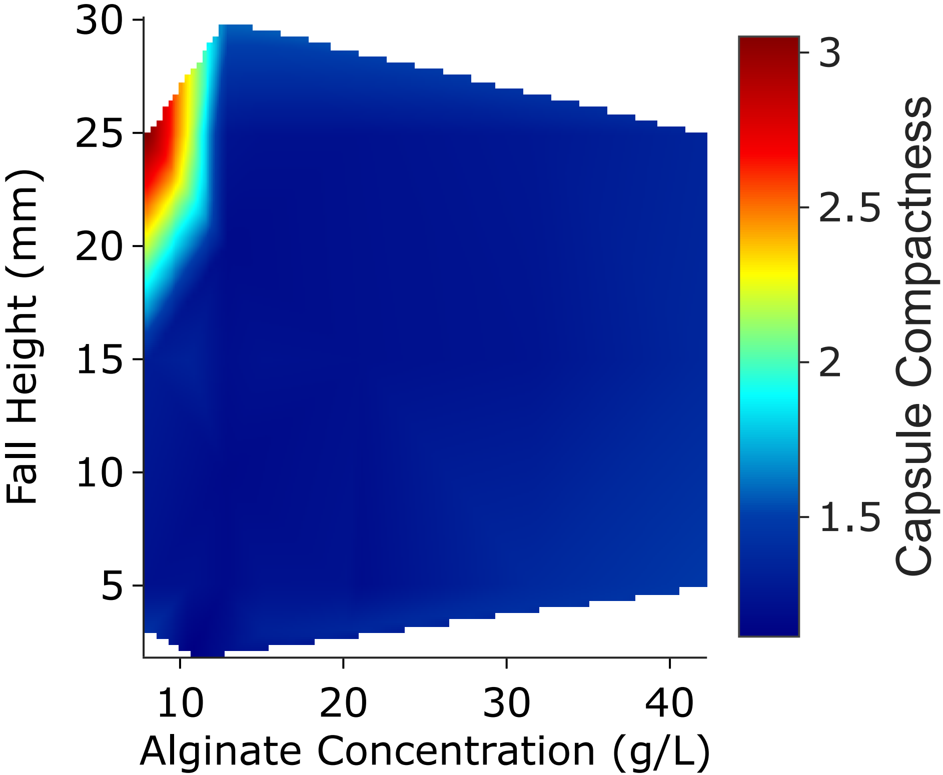

30
25
20
15
10
5
Fall Height (mm)
10
20
30
40
Alginate Concentration (g/L)
3
2.5
2
1.5
Capsule Compactness
