# Supplementary material for: Effects of process parameters on capsule size and shape in the centrifugal encapsulation technology: Parametric study dataset
Source: Data Brief. 2022 Jan 20;41:107851. doi: 10.1016/j.dib.2022.107851 (PMC8804192; doi:10.1016/j.dib.2022.107851)
Supplement: Supplementary file 1 [file mmc1.zip › Word/images.zip!/device_geometry_v8.pdf]

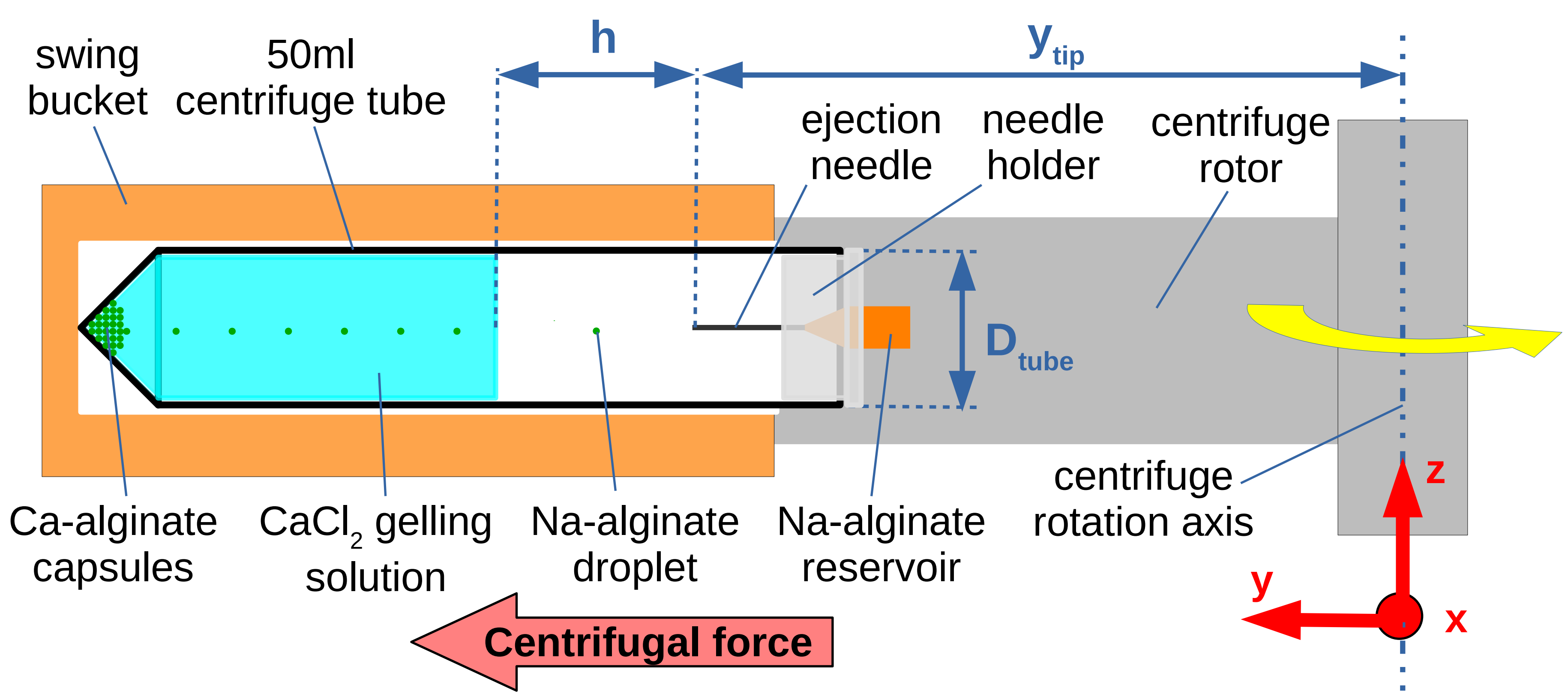
h
y_{tip}
swing bucket
50ml centrifuge tube
ejection needle
needle holder
centrifuge rotor
D_{tube}
centrifuge rotation axis
z
Ca-alginate capsules
$CaCl_2$ gelling solution
Na-alginate droplet
Na-alginate reservoir
y
x
Centrifugal force
