# Supplementary material for: Effects of process parameters on capsule size and shape in the centrifugal encapsulation technology: Parametric study dataset
Source: Data Brief. 2022 Jan 20;41:107851. doi: 10.1016/j.dib.2022.107851 (PMC8804192; doi:10.1016/j.dib.2022.107851)
Supplement: Supplementary file 1 [file mmc1.zip › Word/images.zip!/diameter.pdf]

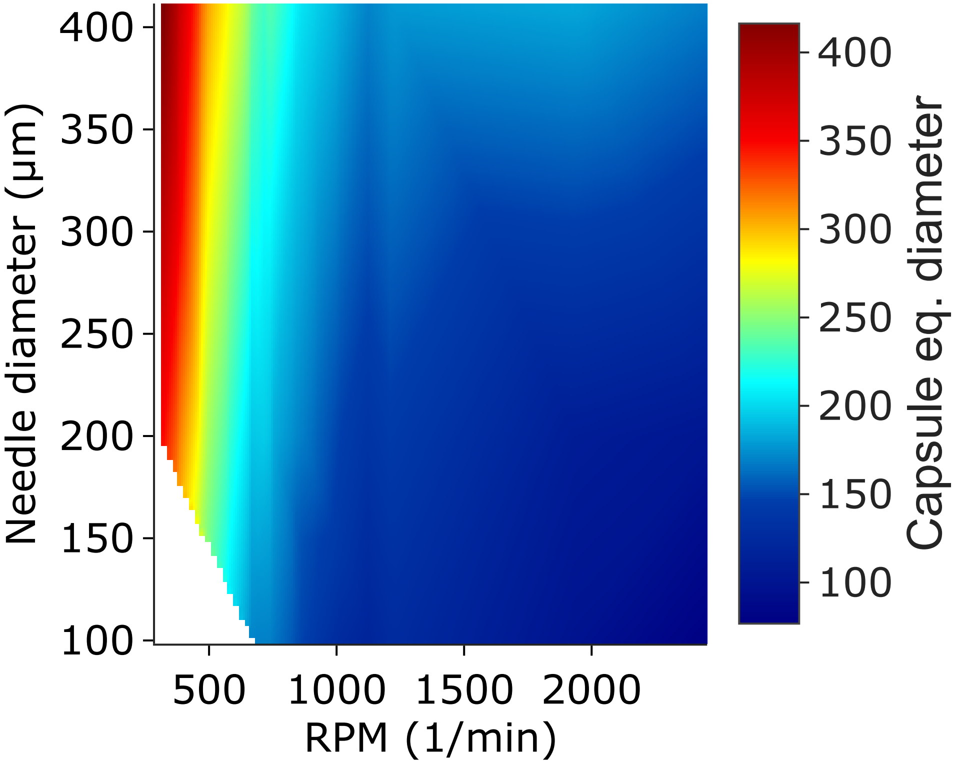

400
350
300
250
200
150
100
Needle diameter (µm)
500
1000
1500
2000
RPM (1/min)
400
350
300
250
200
150
100
Capsule eq. diameter
