# Supplementary material for: Effects of process parameters on capsule size and shape in the centrifugal encapsulation technology: Parametric study dataset
Source: Data Brief. 2022 Jan 20;41:107851. doi: 10.1016/j.dib.2022.107851 (PMC8804192; doi:10.1016/j.dib.2022.107851)
Supplement: Supplementary file 1 [file mmc1.zip › Word/images.zip!/position_tubes_v2.pdf]

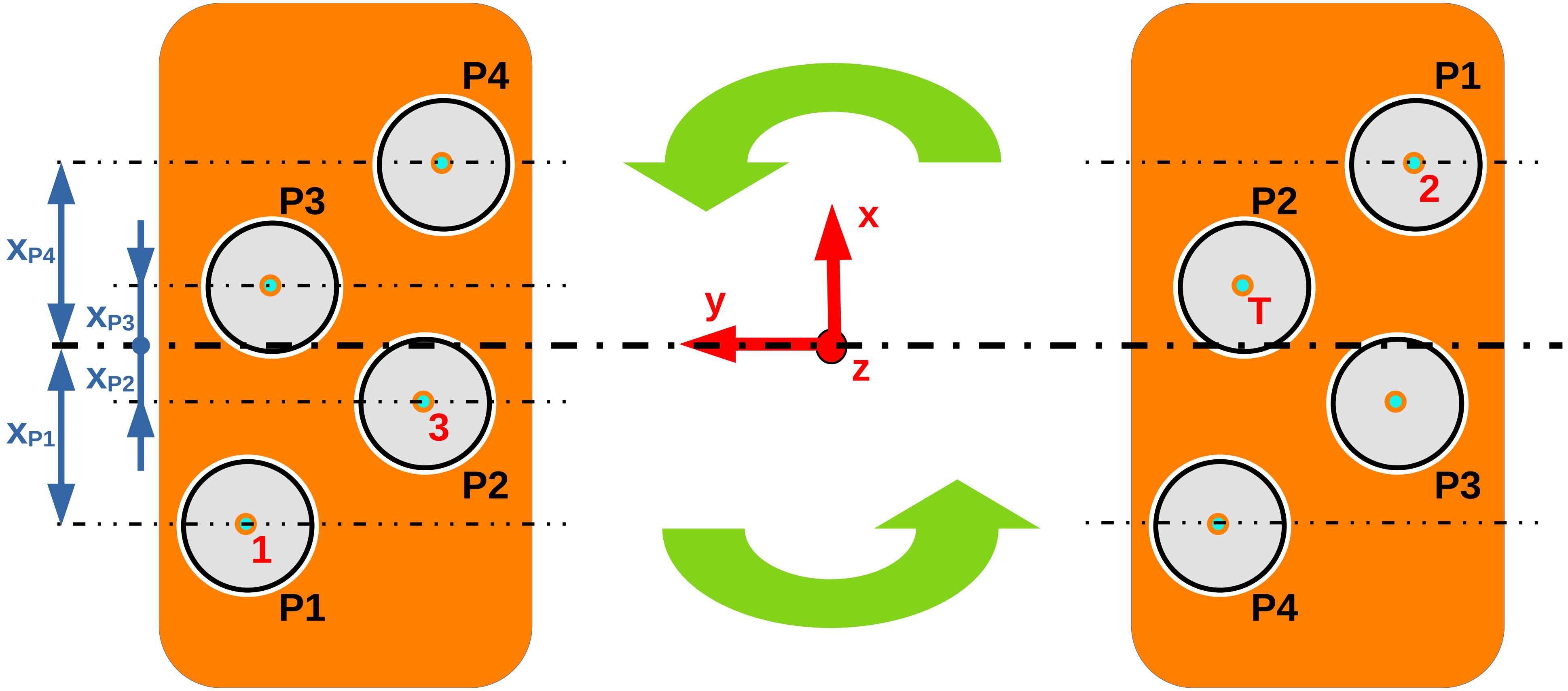

P4
P3
P2
P1
x_{P4}
x_{P3}
x_{P2}
x_{P1}
3
1
x
y
z
P1
P2
2
T
P3
P4
