# Supplementary material for: Effects of process parameters on capsule size and shape in the centrifugal encapsulation technology: Parametric study dataset
Source: Data Brief. 2022 Jan 20;41:107851. doi: 10.1016/j.dib.2022.107851 (PMC8804192; doi:10.1016/j.dib.2022.107851)
Supplement: Supplementary file 1 [file mmc1.zip › Word/images.zip!/solidity.pdf]

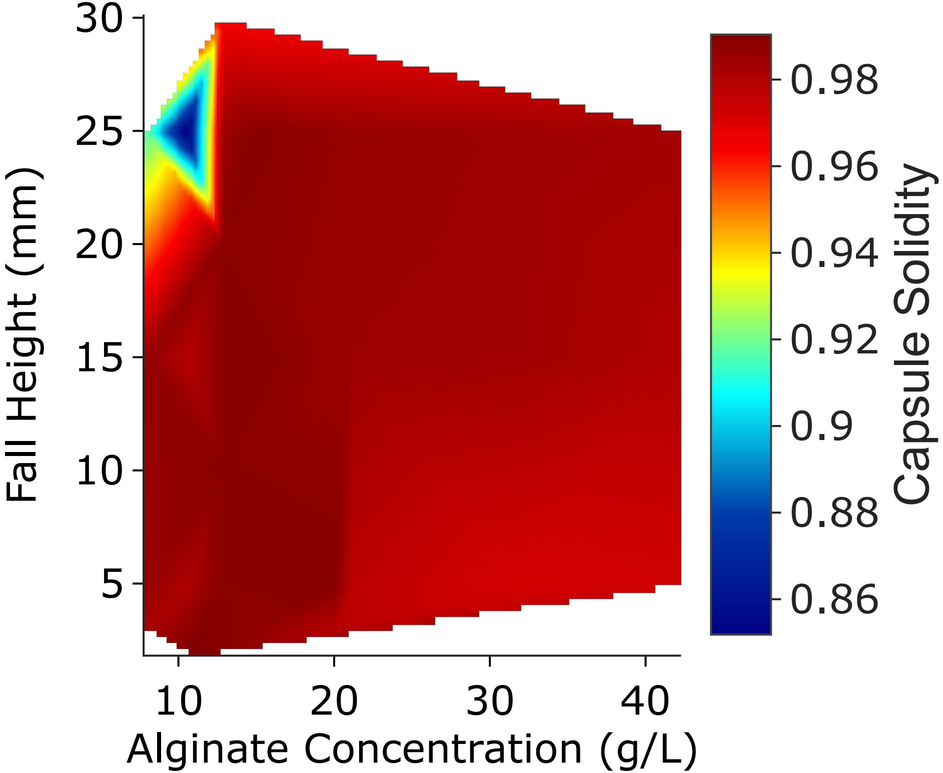

Fall Height (mm)
30
25
20
15
10
5
10
20
30
40
Alginate Concentration (g/L)
0.98
0.96
0.94
0.92
0.9
0.88
0.86
Capsule Solidity
